# Ammonium Pyrrolidine Dithiocarbamate-Modified CdTe/CdS Quantum Dots as a Turn-on Fluorescent Sensor for Detection of Trace Cadmium Ions

**DOI:** 10.3390/s20010312

**Published:** 2020-01-06

**Authors:** Yuan Yin, Qingliang Yang, Gang Liu

**Affiliations:** 1 Key Lab of Modern Precision Agriculture System Integration Research, Ministry of Education of China, China Agricultural University, Beijing 100083, China; b20173080592@cau.edu.cn (Y.Y.); qingliangyang@cau.edu.cn (Q.Y.); 2 Key Lab of Agriculture Information Acquisition Technology, Ministry of Agriculture of China, China Agricultural University, Beijing 100083, China

**Keywords:** quantum dot, cadmium, ammonium pyrrolidine dithiocarbamate, fluorescence quenching, fluorescent sensor

## Abstract

In this work, ammonium pyrrolidine dithiocarbamate (APDC) was used as a surface etchant to modify CdTe/CdS core-shell quantum dots (QDs). The APDC etchant combines with the cadmium ions (Cd^2+^) on the surface of the QDs, resulting in the formation of surface holes. The formation of these holes changes the QD surface structure, which leads to fluorescence quenching of the QDs. Newly added Cd^2+^ can selectively recognize and combine with these holes; thus, the fluorescence intensity of the QDs can be restored. The linear response of this turn-on fluorescent sensor was found to be 0–100 μg/L and 100–600 μg/L under the determined optimal conditions, and its limit of detection (LOD) for Cd^2+^ was 2.642 μg/L (23.5 nmol/L).

## 1. Introduction

Cadmium is a highly toxic heavy metal with a long biological half-life, easily accumulates in organisms, and is difficult to eliminate [1,2,3]. The International Agency for Research on Cancer (IARC) has defined cadmium as a human carcinogen that can lead to liver cancer, kidney cancer, and osteomalacia, and cadmium is a soil and water contaminant strictly controlled by all countries [4,5,6]. Therefore, due to the human health effects and pollution risk of cadmium, its detection in the environment, especially its rapid detection, is of great significance [7,8,9].

The need for cadmium detection has increased over the past few years, and the methods commonly used for cadmium detection are atomic absorption spectrometry (AAS) [10,11], atomic emission spectrometry (AES) [12,13], inductively coupled plasma mass spectrometry (ICPMS) [14,15], conductometric analysis (CA) [16,17], anodic stripping voltammetry (ASV) [18,19,20,21], immunoassays, and biosensors. AAS, AES, and ICPMS all require large-scale analysis equipment and complex operations. CA and ASV involve a long reaction or deposition process of more than 5 min, and both require a complex electrode modification procedure. Biosensors require strict storage conditions to prevent their invalidation [22,23,24,25]. The disadvantages of these methods limit their application for portable and rapid detection of trace Cd^2+^.

Quantum dots (QDs) are inorganic fluorescent clusters that have been developed in recent years and have unique optical properties, such as an adjustable size and emission wavelength, strong fluorescence stability, and high fluorescence efficiency. Compared with the cadmium detection methods mentioned above, fluorescent QD sensors are increasingly used for biomolecule and metal ion (such as Cd^2+^, Cu^2+^, Pb^2+^, Hg^2+^, and Ag^+^) detection due to their small size, simple usage, good preservation, and good selectivity [26,27,28,29].

**Figure 1:**
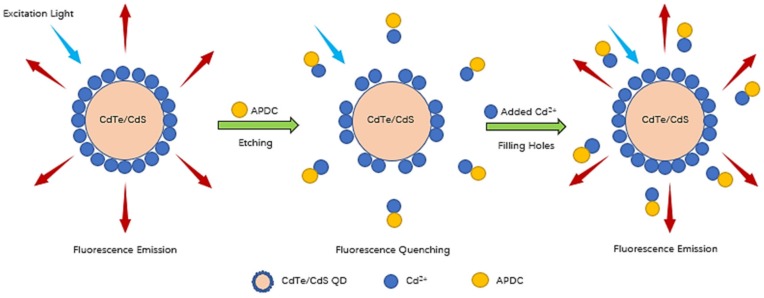
The reaction mechanism of the turn-on mode CdTe/CdS quantum dots (QD) sensor.

At present, the QD sensors commonly used for cadmium measurement are based on Cd^2+^, especially cadmium telluride (CdTe) and cadmium selenide (CdSe) QDs, and are all single-core QDs [30,31,32]. Most of these reported QDs use the turn-off mode for Cd^2+^ detection; only a few use the turn-on mode, which has a lower false positive rate than the turn-off mode and a limit of detection (LOD) in the range of 11–62 μg/L (0.097–0.552 μmol/L) [33,34,35]. In this work, a simple CdTe/CdS core-shell QD sensor that uses the turn-on mode was developed for portable and rapid detection of Cd^2+^, and compared with the single-core QD structure, the core-shell structure leads to stronger fluorescence stability, higher fluorescence efficiency, and lower toxicity. This sensor is formed by using ammonium pyrrolidine dithiocarbamate (APDC) to etch the surface of CdTe/CdS core-shell QDs, which leads to the formation of holes on the QD surface that cause fluorescence quenching. Cd^2+^ ions selectively recognize and combine with these holes, leading to recovery of the QD fluorescence. The reaction mechanism of this turn-on-mode QD sensor is shown in Figure 1.

## 2. Experimental

### 2.1. Instruments and Reagents

All measurement processes were performed using a ThinkPad X230 notebook computer (Lenovo, Beijing, China), a USB4000-XR1-ES modular spectrometer with related software and an optical fiber (Ocean Optics, Dunedin, USA), an LED460-T optical source (Wyoptics Technology, Shanghai, China), and a 4.5-mL UV cuvette (Fisher Scientific, Shanghai, China). All reagent treatment processes involved the use of a BSA224S electronic balance (Satorius Group, Gottingen, Germany) and a Research Plus pipette (Eppendorf, Shanghai, China). Ultrapure water was prepared using a ultrapure water system (model: UPT-UPHW) (Ulupure Ultrapure Technology, Chengdu, China).

CdTe/CdS core-shell QDs were purchased from Xingzi New Material Technology Development (Shanghai, China). Tris-HCl buffer solution and APDC reagent were purchased from Senbeijia Biological Technology (Nanjing, China). The cadmium solution standard was purchased from Boyao Biological Technology (Shanghai, China). Sodium nitrate, potassium nitrate, aluminum nitrate, calcium nitrate, magnesium nitrate, chromic nitrate, manganous nitrate, ferric chloride, silver nitrate, copper sulfate, mercury nitrate, lead nitrate, and zinc nitrate were all obtained from Macklin Biochemical Technology (Shanghai, China). Ultrapure water (18.24 MΩ/cm) was used in all experiments.

### 2.2. Fluorescence Intensity Measurements

In the initial fluorescence intensity measurement, 2 mL of 10 mmol/L Tris-HCl buffer solution with a pH of 8.5 was injected into a 4.5-mL cuvette. Then, 0.4 mL of 5 μmol/L CdTe/CdS QD solution was added to the cuvette and 1.6 mL of the same Tris-HCl buffer solution was added to bring the volume of solution in the cuvette to 4 mL. Finally, the cuvette was placed into the spectrometer and the fluorescence intensity was measured under excitation at 460 nm.

To determine the optimal volume of APDC solution to add to the cuvette, the first 2 steps were the same as those in the initial measurement. In the 3rd step, different volumes of 100 μmol/L APDC solution were added into different cuvettes to etch the QDs and to induce QD fluorescence quenching. Then, 1 μL of 1 g/L cadmium standard solution was added to these cuvettes and the volume of solution in the cuvettes was brought to 4 mL with the same buffer solution used in the initial measurement. Finally, the cuvettes were placed in the spectrometer and their fluorescence intensities were measured under the same excitation conditions mentioned above.

After the optimal volume of APDC solution volume was determined, the detection time and pH of the buffer solution were optimized while holding the APDC solution volume and other conditions constant to determine the optimal detection conditions.

Under these optimal experimental conditions, the volume of the 1 g/L cadmium standard solution needed to produce a signal that fell on the fluorescence intensity curve of the etched QD solution for Cd^2+^ detection was determined, and then, different interfering ions were added to the etched QD solution to investigate the detection robustness. Finally, the fluorescence detection by the etched QD solution was verified by real sample analysis.

## 3. Results and Discussion

### 3.1. The Stability of the Fluorescence Intensity of the QD Solution

**Figure 2:**
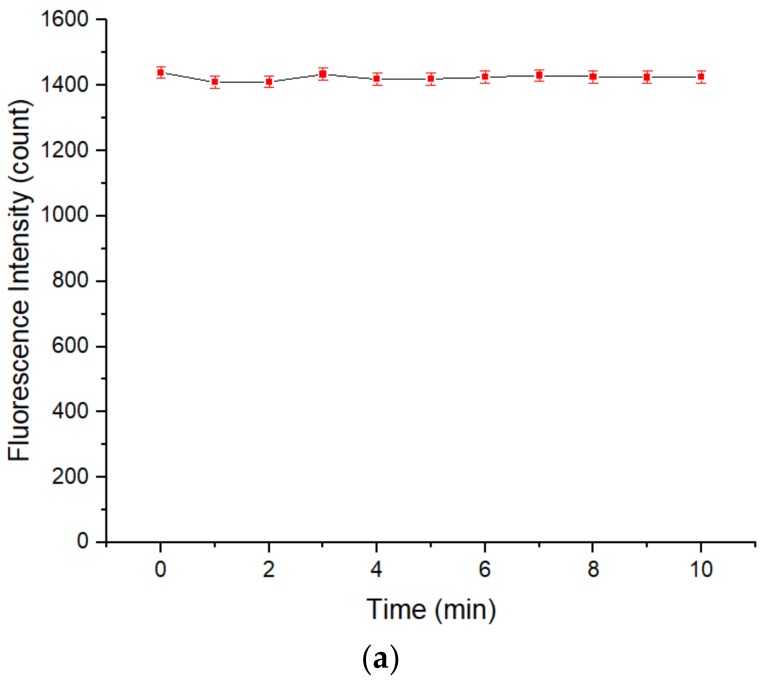

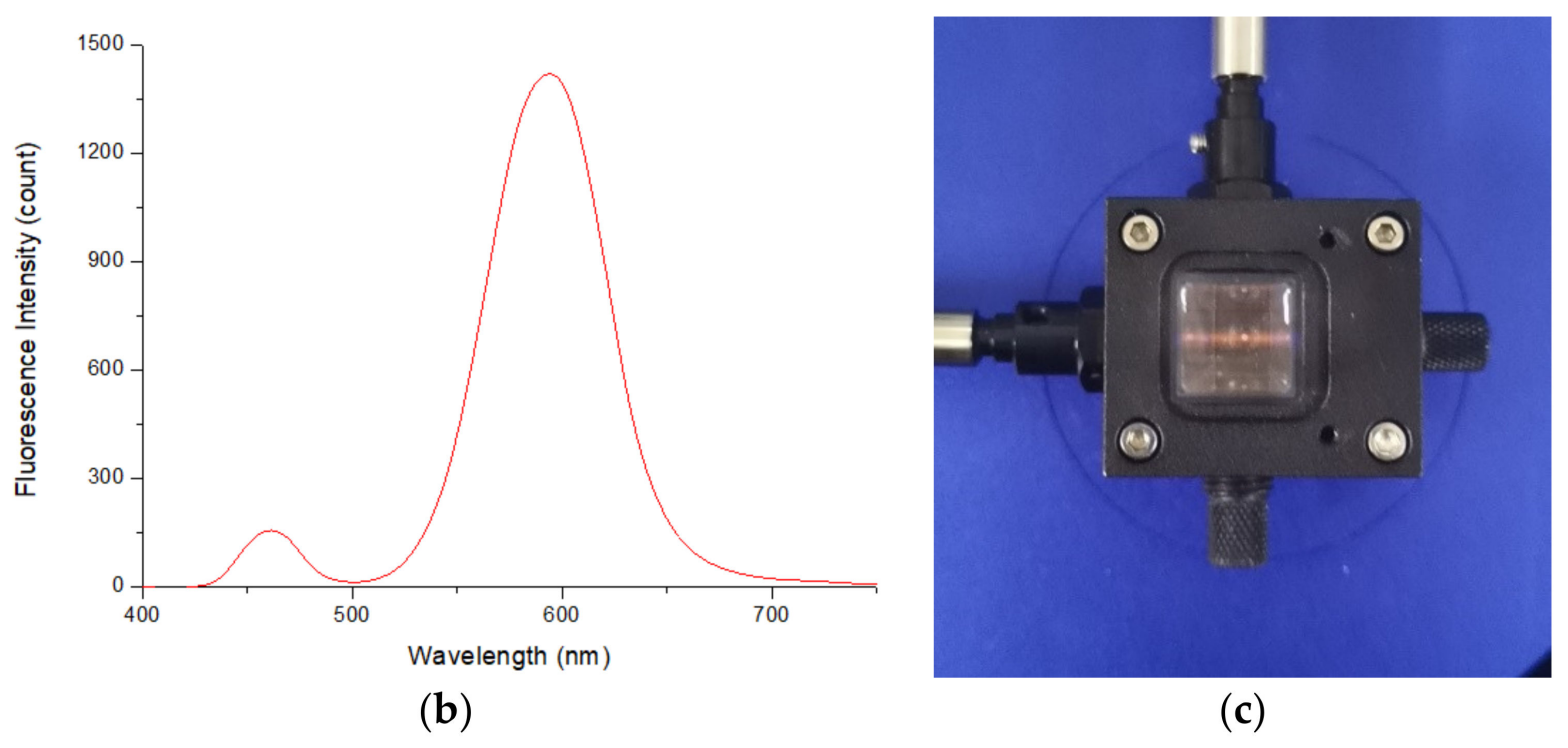
(**a**) Relationship between the fluorescence intensity of a QD solution without etching and time. Concentration of Tris-HCl buffer solution: 10 mmol/L. pH of Tris-HCl buffer solution: 8.5. Concentration of added QD solution: 5 μmol/L. Addition order: Tris-HCl buffer solution (2 mL) → QD solution (0.4 mL) → Tris-HCl buffer solution (1.6 mL). Excitation wavelength: 460 nm. Observation time: 10 min. (**b**,**c**) The fluorescence response of a QD solution without etching. Concentration of Tris-HCl buffer solution: 10 mmol/L. pH of Tris-HCl buffer solution: 8.5. Concentration of added QD solution: 5 μmol/L. Addition order: Tris-HCl buffer solution (2 mL) → QD solution (0.4 mL) → Tris-HCl buffer solution (1.6 mL). Excitation wavelength: 460 nm. All data in this figure is mean of five measurements.

To investigate the stability of the fluorescence intensity of the QD solution, the fluorescence intensity of a QD solution without etching was measured from 0 to 10 min. As shown in Figure 2a, the variation in the fluorescence intensity of the QD solution was less than 0.85% over the detection time range, showing that the fluorescence intensity of the QD solution did not significantly vary over time and that the QD solution has good stability and a long fluorescence lifetime. Figure 2b shows the fluorescence intensity response curve of the QD solution without etching. The initial fluorescence intensity of this solution was 1420.25, and there are 2 emission peaks with wavelengths of 461.13 nm and 594.19 nm. The fluorescence peak near 594.19 nm may represent the fluorescence emitted by the core and shell of the QDs upon excitation. Because the change in the QD shell structure results in a change in the fluorescence intensity of the QD solution, the fluorescence peak intensity near 594.19 nm is used as the index to express the fluorescence intensity of the QD solution in the following discussion. As shown in Figure 2c, the QD solution in the cuvette emitted a bright orange band under excitation at 460 nm.

### 3.2. Optimization of the Concentration of APDC in the Detection Cuvette

The concentration of APDC in solution affects the number of holes etched on the QD surface, and the number of these holes affects the performance of the QD solution for Cd^2+^ detection. To optimize the concentration of APDC in solution, different volumes of 100 μmol/L APDC solution were added to cuvettes containing QD solution at the same volume and concentration, and the fluorescence intensity of the QD solution was measured after addition of the APDC solution. Then, the same volume of 1 g/L cadmium standard solution was added to these solutions and the fluorescence intensity was measured again. The fluorescence intensity recovery ratio was calculated by dividing the fluorescence intensity after addition of the cadmium standard solution by the fluorescence intensity before addition of the cadmium standard solution.

**Figure 3:**
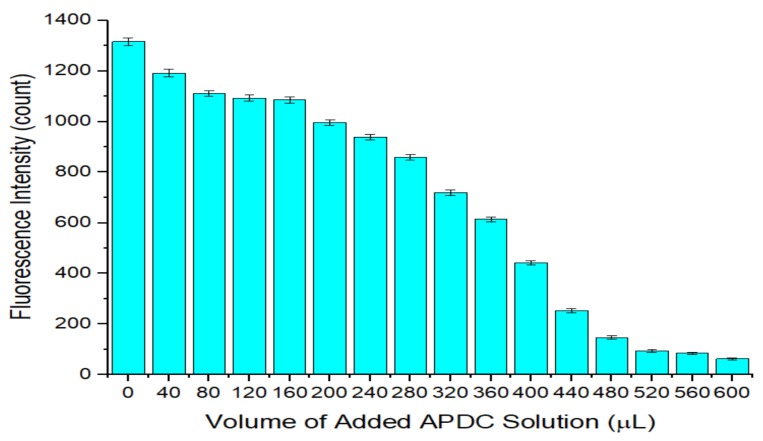
Relationship between the fluorescence intensity of the QD solution and the volume of added ammonium pyrrolidine dithiocarbamate (APDC) solution. Concentration of Tris-HCl buffer solution: 10 mmol/L. pH of Tris-HCl buffer solution: 8.5. Concentration of added QD solution: 5 μmol/L. Concentration of added APDC solution: 100 μmol/L. Addition order: Tris-HCl buffer solution (2 mL) → QD solution (0.4 mL) → APDC solution (0–0.6 mL) → Tris-HCl buffer solution (1.6–1 mL). Excitation wavelength: 460 nm. Observation time: 3 min. All data in this figure is mean of five measurements.

As shown in Figure 3, as the volume of APDC solution increased, the fluorescence intensity of the QD solution gradually decreased because the QD surface contains complexes of cadmium and mercaptan, which act as a passivation layer to eliminate the surface defects in the QD, enabling the QD to emit fluorescence upon excitation. However, as a metal-chelating agent, APDC can combine with Cd^2+^ on the QD surface to destroy the passivation layer. The QD surface is etched because of the loss of Cd^2+^, forming holes. These holes are considered new defects on the QD surface and lead to a decrease in the fluorescence intensity of the QD, namely, fluorescence quenching. As more APDC solution is added, more holes are produced on the QD surface and the fluorescence intensity of the QDs decreases.

**Figure 4:**
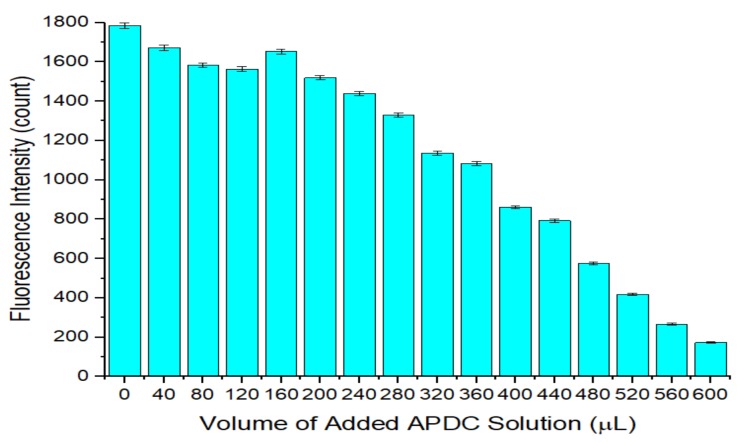
Relationship between the fluorescence intensity of the QD solution with added cadmium standard and the volume of the added APDC solution. Concentration of Tris-HCl buffer solution: 10 mmol/L. pH of Tris-HCl buffer solution: 8.5. Concentration of added QD solution: 5 μmol/L. Concentration of added APDC solution: 100 μmol/L. Concentration of cadmium standard solution: 1 g/L. Addition order: Tris-HCl buffer solution (2 mL) → QD solution (0.4 mL) → APDC solution (0–0.6 mL) → cadmium standard solution (0.001 mL) → Tris-HCl buffer solution (1.6–1 mL). Excitation wavelength: 460 nm. Observation time: 3 min. All data in this figure is mean of five measurements.

Figure 4 indicates that the newly added Cd^2+^ fills some holes on the QD surface, which reduces the surface defects and restores the fluorescence intensity. However, as the amount of added APDC solution increases, the newly added Cd^2+^ will increasingly combine with APDC to form chelates. This mechanism competes with the process of filling holes on the QD surface by Cd^2+^, which hinders repair of surface defects by newly added Cd^2+^. The higher the concentration of APDC is, the stronger the hindrance, the fewer the surface defects repaired, and the lower the fluorescence intensity recovered by the QD solution.

**Figure 5:**
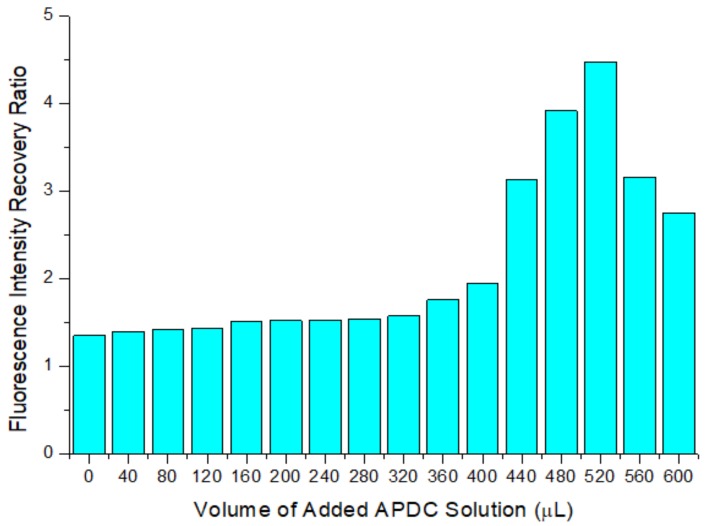
The fluorescence intensity recovery ratio of the etched QD solution with added cadmium standard. Concentration of Tris-HCl buffer solution: 10 mmol/L. pH of Tris-HCl buffer solution: 8.5. Concentration of added QD solution: 5 μmol/L. Concentration of added APDC solution: 100 μmol/L. Concentration of cadmium standard solution: 1 g/L. Addition order: Tris-HCl buffer solution (2 mL) → QD solution (0.4 mL) → APDC solution (0–0.6 mL) → cadmium standard solution (0.001 mL) → Tris-HCl buffer solution (1.6–1 mL). Excitation wavelength: 460 nm. All data in this figure is mean of five measurements.

The fluorescence intensity recovery ratio indicates the Cd^2+^ detection ability of the etched QD solution. The higher the ratio is, the easier it is for the etched QD solution to be repaired by the newly added Cd^2+^ to restore the fluorescence intensity. As shown in Figure 5, with an increase in the amount of APDC solution added, the recovery ratio gradually increases. When 520 μL of APDC solution was added, the recovery ratio reached a maximum of 4.483. When the amount of APDC solution added continued to increase, the ratio began to decrease. This is because, when too little APDC solution is added, few holes are etched on the QD surface, the newly added Cd^2+^ can fill the holes, and the fluorescence intensity recovery ratio of the QD solution is low. However, when there is too much APDC solution, the number of etched holes on the surface of the QDs increases significantly, but compared with the number of Cd^2+^ ions filling these holes, most of the newly added Cd^2+^ combines with APDC to form complexes, which hinders the QD surface defect repair process and reduces the fluorescence intensity recovery ratio of the QD solution. Therefore, the optimal concentration of APDC in the mixed solution was 13 μmol/L, and the optimal volume of added APDC solution was 520 μL.

### 3.3. Optimization of the pH of the Tris-HCl Buffer Solution

**Figure 6:**
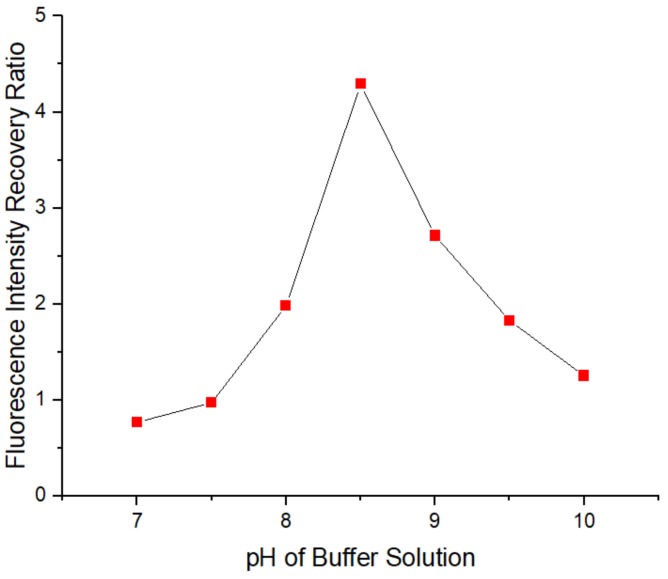
The relationship between the fluorescence intensity recovery ratio and the pH of Tris-HCl buffer solution. Concentration of Tris-HCl buffer solution: 10 mmol/L. Concentration of added QD solution: 5 μmol/L. Concentration of added APDC solution: 100 μmol/L. Concentration of cadmium standard solution: 1 g/L. Addition order: Tris-HCl buffer solution (2 mL) → QD solution (0.4 mL) → APDC solution (0.52 mL) → cadmium standard solution (0.001 mL) → Tris-HCl buffer solution (1.08 mL). Excitation wavelength: 460 nm. All data in this figure is mean of five measurements.

Figure 6 shows the relationship between the fluorescence intensity recovery ratio and the pH of the Tris-HCl buffer solution. With increasing pH, the recovery ratio increased, and when the pH of the Tris-HCl buffer solution was 8.5, the recovery ratio reached a maximum of 4.291. When the pH of the buffer solution continued to increase, the ratio began to decrease. This phenomenon is likely due to the instability and decomposition of the cadmium and mercaptan complexes on the surface of the QDs when the pH of the buffer solution was low, leading to an increase in the number of holes on the surface of the QDs and a decrease in the recovery ratio when the number of newly added Cd^2+^ ions is constant. At high pH, the buffer solution contains abundant OH– ions, which may preferentially combine with the newly added Cd^2+^ to form Cd(OH)_2_, reducing the number of newly added Cd2+ ions to fill the holes on the QD surface and reducing the fluorescence intensity recovery ratio of the QD solution. Consequently, the optimal pH of the Tris-HCl buffer solution was 8.5.

### 3.4. Fluorescence Quenching Caused by the Etching Effect of APDC in the CdTe/CdS QD Solution

**Figure 7:**
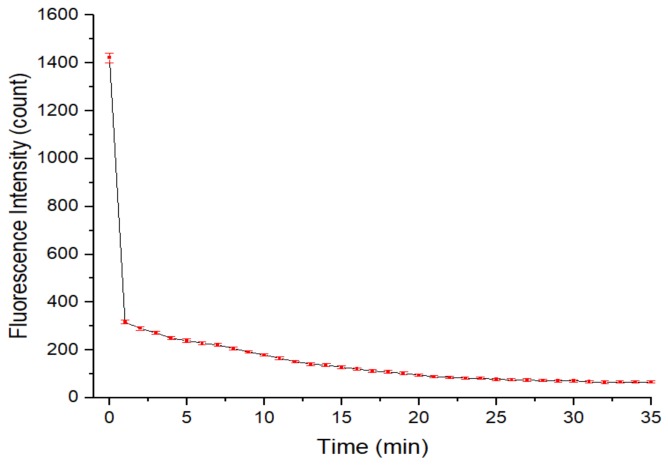
Relationship between the fluorescence intensity of the QD solution and the time after addition of the APDC solution. Concentration of Tris-HCl buffer solution: 10 mmol/L. pH of Tris-HCl buffer solution: 8.5. Concentration of added QD solution: 5 μmol/L. Concentration of APDC solution: 100 μmol/L. Addition order: Tris-HCl buffer solution (2 mL) → QD solution (0.4 mL) → APDC solution (0.52 mL) → Tris-HCl buffer solution (1.08 mL). Excitation wavelength: 460 nm. Observation time: 35 min. All data in this figure is mean of five measurements.

To investigate the fluorescence quenching caused by APDC etching in the QD solution, after addition of the APDC solution to the QD solution in the cuvette, the fluorescence intensity of the QD solution was measured. As shown in Figure 7, the fluorescence of the QD solution was quenched in the first minute after addition of the APDC solution and the fluorescence intensity of the QD solution decreased rapidly by 77.68%. This phenomenon shows that APDC caused rapid damage to the surface structure of the QDs, and the fluorescence intensity of the QD solution subsequently decreased slowly. After the 25th minute, the fluorescence intensity no longer changed significantly. Therefore, 25 min was chosen to prepare the APDC/(CdTe/CdS) blank contrast solution without added Cd^2+^ for comparison and analysis in the subsequent measurement process.

### 3.5. Fluorescence Recovery Caused by Addition of Cd^2+^ to the APDC/CdTe/CdS QD Solution

**Figure 8:**
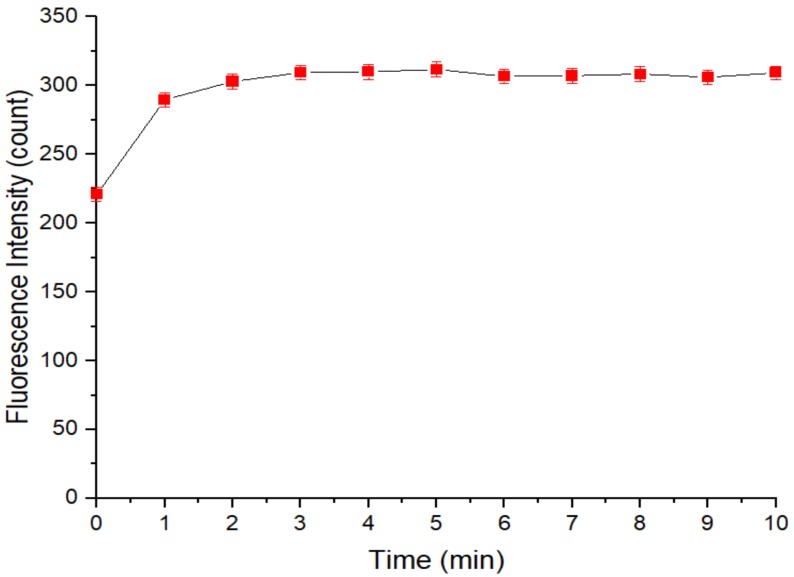
Relationship between the fluorescence intensity of the restored QD solution and the time after addition of the cadmium standard solution. Concentration of Tris-HCl buffer solution: 10 mmol/L. pH of Tris-HCl buffer solution: 8.5. Concentration of added QD solution: 5 μmol/L. Concentration of APDC solution: 100 μmol/L. Concentration of cadmium standard solution: 1 g/L. Addition order: Tris-HCl buffer solution (2 mL) → QD solution (0.4 mL) → APDC solution (0.52 mL) → cadmium standard solution (0.001 mL) → Tris-HCl buffer solution (1.08 mL). Excitation wavelength: 460 nm. Observation time: 10 min. All data in this figure is mean of five measurements.

Figure 8 depicts the relationship between the fluorescence intensity of the restored QD solution and the time after addition of the cadmium standard solution. The fluorescence intensity of the restored QD solution recovered to 220.99 after the initial measurement and then to 289.75 after 1 min. After the second minute, the fluorescence intensity of the solution no longer changed significantly, which meant that some of the surface holes had been filled rapidly in these 2 min, and the fluorescence intensity of the QD solution was restored to a certain extent. The average fluorescence intensity of the QD solution was 308.02 from the 3rd to 10th minute, and the average fluorescence intensity recovery ratio of the QD solution was 4.569, which is almost consistent with the fluorescence intensity recovery ratio corresponding to the optimal addition amount of APDC solution mentioned above.

### 3.6. Analytical Performance of the APDC/CdTe/CdS QD Solution

**Figure 9:**
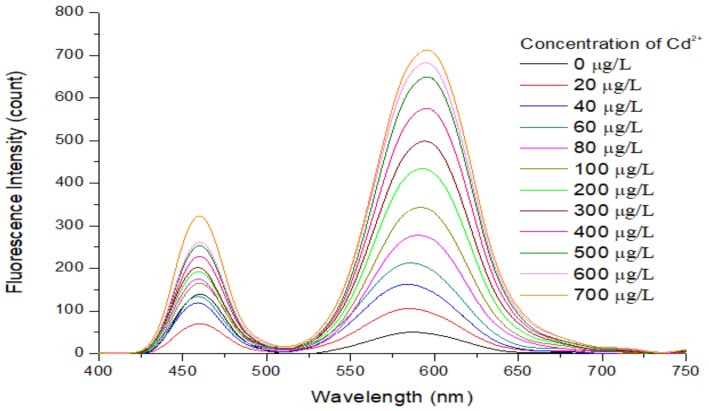
Relationship between the fluorescence intensity of the restored QD solution and the concentration of added Cd^2+^ in the QD solution. Concentration of Tris-HCl buffer solution: 10 mmol/L. pH of Tris-HCl buffer solution: 8.5. Concentration of added QD solution: 5 μmol/L. Concentration of APDC solution: 100 μmol/L. Concentration of cadmium standard solution: 1 g/L. Addition order: Tris-HCl buffer solution (2 mL) → QD solution (0.4 mL) → APDC solution (0.52 mL) → cadmium standard solution (0–0.0028 mL) → Tris-HCl buffer solution (1.08–1.0772 mL). Excitation wavelength: 460 nm. Observation time: 3 min. All data in this figure is mean of five measurements.

**Figure 10:**
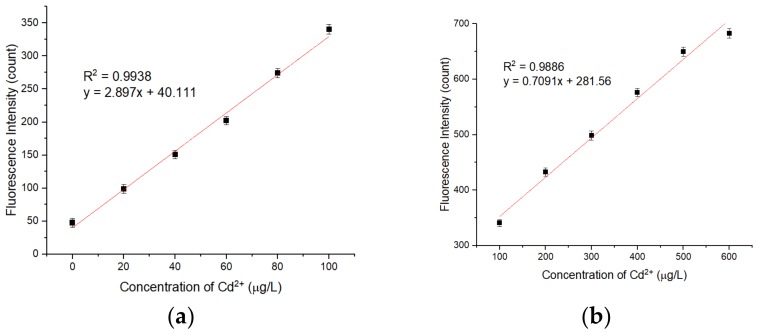
(**a**) Corresponding calibration plots for the APDC/CdTe/CdS QD solution based on different Cd^2+^ concentrations in Tris-HCl buffer solution. Concentration of Tris-HCl buffer solution: 10 mmol/L. pH of Tris-HCl buffer solution: 8.5. Concentration of added QD solution: 5 μmol/L. Concentration of APDC solution: 100 μmol/L. Concentration of cadmium standard solution: 1 g/L. Addition order: Tris-HCl buffer solution (2 mL) → QD solution (0.4 mL) → APDC solution (0.52 mL) → cadmium standard solution (0–0.0004 mL) → Tris-HCl buffer solution (1.08 mL). Excitation wavelength: 460 nm. Observation time: 3 min. * Mean of five measurements. (**b**) Corresponding calibration plots for the APDC/CdTe/CdS QD solution based on different Cd^2+^ concentrations in Tris-HCl buffer solution. Concentration of Tris-HCl buffer solution: 10 mmol/L. pH of Tris-HCl buffer solution: 8.5. Concentration of added QD solution: 5 μmol/L. Concentration of APDC solution: 100 μmol/L. Concentration of cadmium standard solution: 1 g/L. Addition order: Tris-HCl buffer solution (2 mL) → QD solution (0.4 mL) → APDC solution (0.52 mL) → cadmium standard solution (0.0004–0.0024 mL) → Tris-HCl buffer solution (1.08–1.0776 mL). Excitation wavelength: 460 nm. Observation time: 3 min. All data in this figure is mean of five measurements.

As shown in Figure 9, the relationship between the fluorescence intensity of the restored QD solution and the concentration of added Cd^2+^ in the QD solution was investigated. Under the optimal detection conditions, the fluorescence intensity of the restored QD solution had a good linear relationship with the concentration of Cd^2+^ in the ranges of 0–100 μg/L and 100–600 μg/L. At the same time, with an increase in the concentration of Cd^2+^, the fluorescence emission peak wavelength of the QD solution was redshifted, which may be a result of gradual filling of the etched holes on the QD surface and the increasing size of the QDs. Under the optimal detection conditions, the fluorescence intensity of the APDC/CdTe/CdS QD solution was repeatedly measured 20 times without addition of the cadmium standard solution, and the standard deviation was calculated. To obtain the LOD, 3 times the standard deviation was divided by the slope of the calibration curve shown in Figure 10a. Similarly, 10 times the standard deviation was divided by the slope of the calibration curve in Figure 10a to obtain the limit of quantitation (LOQ). The LOD of the APDC/CdTe/CdS QD solution for Cd^2+^ detection was 2.642 μg/L (23.5 nmol/L), the LOQ of the APDC/CdTe/CdS QD solution for Cd^2+^ detection was 8.807 μg/L (78.4 nmol/L), and the detection time was less than 3 min. As shown in Figure 10a, the equation *y* = 2.897*x* + 40.111 (y: count, *x*: μg/L) with a correlation coefficient of 0.9938 (S/N = 3) was obtained for a Cd^2+^ concentration range of 0–100 μg/L, and Figure 10b shows that the equation *y* = 0.7091*x* + 281.56 (*y*: count, *x*: μg/L) with a correlation coefficient of 0.9886 (S/N = 3) was obtained for a Cd^2+^ concentration range of 100–600 μg/L. Table 1 is the comparison of different QD sensors or fluorescent probes for Cd^2+^ determination.

### 3.7. Interference Study

**Figure 11:**
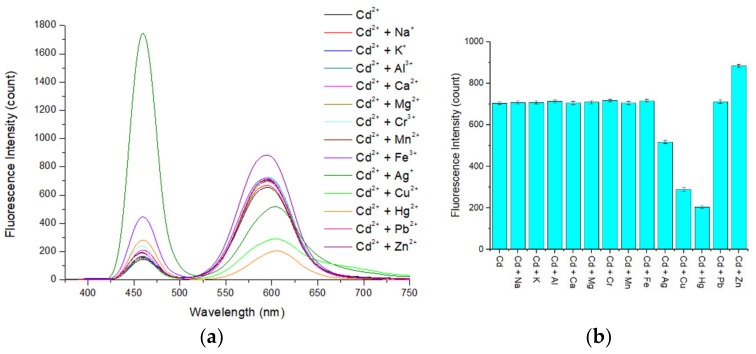
(**a**,**b**) Relationship between the fluorescence intensity of the restored QD solution and the presence of added Cd^2+^ and other ions in the QD solution. Concentration of Tris-HCl buffer solution: 10 mmol/L. pH of Tris-HCl buffer solution: 8.5. Concentration of added QD solution: 5 μmol/L. Concentration of APDC solution: 100 μmol/L. Concentration of cadmium standard solution: 2.5 mmol/L. Concentration of other standard solutions: 2.5 mmol/L. Addition order: Tris-HCl buffer solution (2 mL) → QD solution (0.4 mL) → APDC solution (0.52 mL) → cadmium standard solution (0.008 mL) → other standard solution (0.008 mL) → Tris-HCl buffer solution (1.064 mL). Excitation wavelength: 460 nm. Observation time: 3 min. All data in this figure is mean of five measurements.

**Figure 12:**
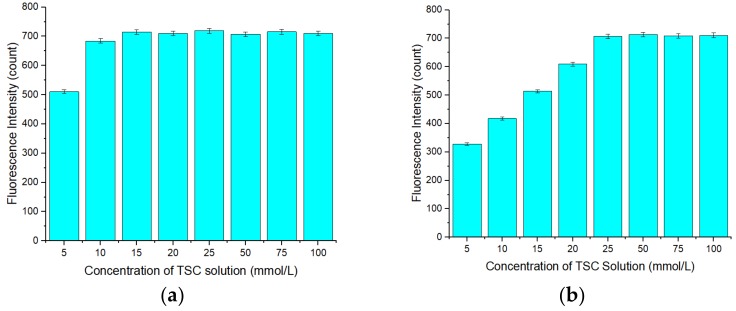
(**a**) Relationship between the fluorescence intensity of the restored QD solution and the concentration of thiosemicarbazide (TSC) solution added into a copper standard solution before detection. Concentration of TSC solution: 5–100 mmol/L. Concentration of copper standard solution before detection: 0.5 mol/L. Mix order: copper standard solution (5 μL) → TSC solution (5 mL). Concentration of Tris-HCl buffer solution: 10 mmol/L. pH of Tris-HCl buffer solution: 8.5. Concentration of added QD solution: 5 μmol/L. Concentration of APDC solution: 100 μmol/L. Concentration of cadmium standard solution: 2.5 mmol/L. Concentration of mixed copper standard solution: 0.5 mmol/L. Concentration of TSC in mixed copper standard solution: 5–100 mmol/L. Addition order: Tris-HCl buffer solution (2 mL) → QD solution (0.4 mL) → APDC solution (0.52 mL) → cadmium standard solution (0.008 mL) → mixed copper standard solution (0.04 mL) → Tris-HCl buffer solution (1.032 mL). Excitation wavelength: 460 nm. Observation time: 3 min. * Mean of five measurements. (**b**) Relationship between the fluorescence intensity of the restored QD solution and the concentration of TSC solution added into a mercury standard solution before detection. Concentration of TSC solution: 5–100 mmol/L. Concentration of mercury standard solution before detection: 0.1 mol/L. Mix order: mercury standard solution (25 μL) → TSC solution (5 mL). Concentration of Tris-HCl buffer solution: 10 mmol/L. pH of Tris-HCl buffer solution: 8.5. Concentration of added QD solution: 5 μmol/L. Concentration of APDC solution: 100 μmol/L. Concentration of cadmium standard solution: 2.5 mmol/L. Concentration of mixed mercury standard solution: 0.5 mmol/L. Concentration of TSC in mixed mercury standard solution: 5–100 mmol/L. Addition order: Tris-HCl buffer solution (2 mL) → QD solution (0.4 mL) → APDC solution (0.52 mL) → cadmium standard solution (0.008 mL) → mixed mercury standard solution (0.04 mL) → Tris-HCl buffer solution (1.032 mL). Excitation wavelength: 460 nm. Observation time: 3 min. All data in this figure is mean of five measurements.

**Figure 13:**
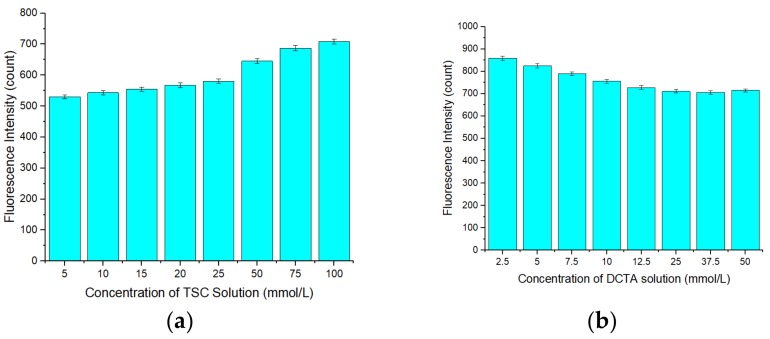
(**a**) Relationship between the fluorescence intensity of the restored QD solution and the concentration of TSC solution added into a silver standard solution before detection. Concentration of TSC solution: 5–100 mmol/L. Concentration of silver standard solution before detection: 0.5 mol/L. Mix order: silver standard solution (5 μL) → TSC solution (5 mL). Concentration of Tris-HCl buffer solution: 10 mmol/L. pH of Tris-HCl buffer solution: 8.5. Concentration of added QD solution: 5 μmol/L. Concentration of APDC solution: 100 μmol/L. Concentration of cadmium standard solution: 2.5 mmol/L. Concentration of mixed silver standard solution: 0.5 mmol/L. Concentration of TSC in mixed silver standard solution: 5–100 mmol/L. Addition order: Tris-HCl buffer solution (2 mL) → QD solution (0.4 mL) → APDC solution (0.52 mL) → cadmium standard solution (0.008 mL) → mixed silver standard solution (0.04 mL) → Tris-HCl buffer solution (1.032 mL). Excitation wavelength: 460 nm. Observation time: 3 min. * Mean of five measurements. (**b**) Relationship between the fluorescence intensity of the restored QD solution and the concentration of (1,2-cyclohexylenedinitrilo)-tetraacetic acid (DCTA) solution added into a zinc standard solution before detection. Concentration of DCTA solution: 2.5–50 mmol/L. Concentration of zinc standard solution before detection: 1 mmol/L. Mix order: DCTA solution (5 mL) → zinc standard solution (5 mL). Concentration of Tris-HCl buffer solution: 10 mmol/L. pH of Tris-HCl buffer solution: 8.5. Concentration of added QD solution: 5 μmol/L. Concentration of APDC solution: 100 μmol/L. Concentration of cadmium standard solution: 2.5 mmol/L. Concentration of mixed zinc standard solution: 0.5 mmol/L. Concentration of DCTA in mixed zinc standard solution: 1.25–25 mmol/L. Addition order: Tris-HCl buffer solution (2 mL) → QD solution (0.4 mL) → APDC solution (0.52 mL) → cadmium standard solution (0.008 mL) → mixed zinc standard solution (0.04 mL) → Tris-HCl buffer solution (1.032 mL). Excitation wavelength: 460 nm. Observation time: 3 min. All data in this figure is mean of five measurements.

To investigate the selectivity of the APDC/CdTe/CdS QD solution, an interference study was conducted. As observed in Figure 11a,b, when the concentration of all ions was 5 μmol/L, the presences of Na^+^, K^+^, Al^3+^, Ca^2+^, Mg^2+^, Cr^3+^, Mn^2+^, Fe^3+^, and Pb^2+^ did not have a significant impact on the fluorescence intensity of the Cd-APDC/CdTe/CdS system. The presence of Ag+, Cu^2+^, and Hg^2+^ led to a decrease in the fluorescence intensity of the system to varying degrees because these ions have a stronger ability than Cd^2+^ to bind the S in mercaptan. At the same time, it is possible that the solubilities of CuTe, Ag2Te, and HgTe are lower than that of CdTe. Thus, when Ag^+^, Cu^2+^, and Hg^2+^ were added, these insoluble compounds easily accumulate on the surface of the QDs, hindering the hole filling by Cd^2+^ and leading to a reduction in the fluorescence intensity recovery of the restored solution and in the fluorescence intensity recovery ratio. When Zn^2+^ is added, the fluorescence intensity of the Cd-APDC/CdTe/CdS system increased, possibly because Zn^2+^ can form compounds similar to Cd-mercaptan on the surface of the QDs, which leads to an increase in the fluorescence intensity of the solution. The fluorescence intensity decrease caused by Ag+, Cu^2+^, and Hg^2+^ was eliminated by addition of the masking agent thiosemicarbazide (TSC) before detection. To investigate the masking effect of TSC for silver, copper, and mercury, a masking effect study was conducted. The silver, copper, and mercury standard solutions were mixed with TSC solution of different concentrations before detection, and then, the mixed silver, copper, and mercury standard solutions were added into the Cd-APDC/CdTe/CdS solution and the fluorescence intensities of these hybrid solutions were measured. As shown in Figure 12a,b and Figure 13a, the fluorescence intensity of the three hybrid solutions increased with an increase in the concentration of the TSC solution added in the pretreatment stage of the silver, copper, and mercury standard solutions. With an increase in the TSC solution concentration, the masking effect became increasingly more obvious and the fluorescence intensities of the hybrid solutions containing mixed silver, copper, and mercury standard solutions returned to the level without interference factors when the concentrations of mixed TSC solution were 15, 25, and 100 mmol/L, respectively. Consequently, the optimal concentration of TSC solution was 100 mmol/L. Similarly, the increase caused by Zn^2+^ was eliminated by addition of (1,2-cyclohexylenedinitrilo)-tetraacetic acid (DCTA) before detection. As can be seen from Figure 13b, the fluorescence intensity of the hybrid solution decreased with an increase in the DCTA solution added in the pretreatment stage of the zinc standard solution. When the concentration of DCTA solution was 50 mmol/L, its masking effect on zinc ions began to reach saturation. According to Figure 13b, the optimal concentration of DCTA solution was 50 mmol/L. According to the above discussion, the APDC/CdTe/CdS QD solution can be used as a selective sensor for Cd^2+^ detection.

### 3.8. Sample Analysis

The feasibility of the APDC/CdTe/CdS QD solution for Cd^2+^ detection was verified by analyzing extracted river water samples. In the pretreatment stage, all samples were mixed with TSC and DCTA solution to mask the interference of Ag+, Cu^2+^, Hg^2+^, and Zn^2+^.

The feasibility of detecting Cd^2+^ in practical samples using the APDC/CdTe/CdS QD solution sensor was verified by analysis of extracted river water samples and comparison with AAS analysis. As shown in Table 2, the relative error of Cd^2+^ detection in river water samples using the APDC/CdTe/CdS QD solution sensor was no more than 3.5%. In addition, Table 3 shows recovery of Cd^2+^ from river water samples using the APDC/CdTe/CdS QD solution sensor. The average recovery for the APDC/CdTe/CdS solution sensor was 98.76%, and the RSD was less than 3%. These results indicate that the APDC/CdTe/CdS QD solution can be used for Cd^2+^ detection in extracted river water samples.

## 4. Conclusions

In this work, a CdTe/CdS QD solution sensor etched by APDC for Cd^2+^ detection is introduced. The sensor is based on the turn-on mode. With the addition of APDC, the surface structure of QDs is etched, which leads to fluorescence quenching. Then, added Cd^2+^ can gradually recover the fluorescence intensity of the APDC/CdTe/CdS QD solution. After optimization of the APDC concentration in the QD solution and the pH, this APDC/CdTe/CdS QD solution can be used as a fluorescent sensor for Cd^2+^ detection in water samples. The LOD of this QD solution sensor is 2.642 μg/L (23.5 nmol/L), and its two linear response ranges are 0–100 μg/L and 100–600 μg/L. In addition, the QD solution sensor has good selectivity, low toxicity, and a fast response time and shows potential for application in environmental Cd^2+^ monitoring.

## Figures and Tables

**Table 1:** Comparison of different QD sensors/fluorescent probes for Cd^2+^ determination.

QD Sensor/Fluorescent Probe	Linear Range (μmol/L)	LOD (μmol/L)	Reference
Ag2S QD	1–40	0.5460	[33]
competitive immunochromatographic strips/gold nanoparticles QD	0.0022–0.0712	0.0016	[34]
4,5-bis (N, N-di (2- hydroxyethyl) iminomethyl) acridine fluorescent probe	1–30	0.1300	[36]
6-mercaptonicotinic acid/L-Cys/gold nanoparticles fluorescent probe	0.2–1.7	0.1000	[37]
CdTe/CdS QD	0.0784–5.338	0.0235	This work

**Table 2:** Comparison of the detection of Cd^2+^ in river water samples using the QD solution sensor and atomic absorption spectrometry (AAS).

Ion	Number	Detected by the QD Solution (μg/L) ^a^	Detected by AAS (μg/L) ^a^	Relative Error (%)	Standard Deviation (SD) (μg/L)	Relative Standard Deviation (RSD) (%)
Cd^2+^	Sample 1	28.75	28.17	2.01	0.56	1.94
	Sample 2	14.39	14.85	3.10	0.41	2.87
	Sample 3	32.81	33.59	2.32	0.77	2.35

^a^ Mean of five measurements. Concentration of TSC solution: 100 mmol/L. Concentration of DCTA solution: 50 mmol/L. Sample mix order: DCTA solution (5 mL)→ water sample (5 mL). Concentration of Tris-HCl buffer solution: 10 mmol/L. pH of Tris-HCl buffer solution: 8.5. Concentration of added QD solution: 5 μmol/L. Concentration of APDC solution: 100 μmol/L. Addition order: Tris-HCl buffer solution (2 mL)→ QD solution (0.4 mL)→ APDC solution (0.52 mL)→ TSC solution (0.04 mL)→ mixed water sample (0.04 mL)→ Tris-HCl buffer solution (1 mL). Excitation wavelength: 460 nm. Observation time: 3 min.

**Table 3:** Recovery of Cd^2+^ from river water samples.

Ion	Number	Added (μg/L)	Detected by the QD Solution (μg/L) ^a.^	Recovery (%)	Standard Deviation (SD) (μg/L)	Relative Standard Deviation (RSD) (%)
Cd^2+^	Sample 4	40	39.10	97.75	0.62	1.77
	Sample 5	80	78.43	98.04	2.08	2.65
	Sample 6	200	202.59	101.3	4.01	1.98
	Sample 7	400	391.73	97.93	9.44	2.41

^a^ Mean of five measurements. Concentration of TSC solution: 100 mmol/L. Concentration of DCTA solution: 50 mmol/L. Sample mix order: DCTA solution (5 mL) → water sample (5 mL). Concentration of Tris-HCl buffer solution: 10 mmol/L. pH of Tris-HCl buffer solution: 8.5. Concentration of added QD solution: 5 μmol/L. Concentration of APDC solution: 100 μmol/L. Concentration of added cadmium standard solution: 40–400 mg/L. Addition order: Tris-HCl buffer solution (2 mL) → QD solution (0.4 mL) → APDC solution (0.52 mL) → cadmium standard solution (0.004 mL) → TSC solution (0.04 mL) → mixed water sample (0.04 mL) → Tris-HCl buffer solution (0.996 mL). Excitation wavelength: 460 nm. Observation time: 3 min.

## References

[1] Fatima G., Raza A.M., Hadi N., Nigam N., Mahdi A.A. (2019). Cadmium in Human Diseases: It’ More than Just a Mere Metal. Indian. J. Biochem. Biol..

[2] Sunli K., Anupama S. (2019). Cadmium toxicity: Effects on human reproduction and fertility. Rev. Environ. Health..

[3] Rebecca M.N., Caterina V.S., Carmen M., Bruce D., Jaymie M.R. (2018). Expression of Genes Involved in Stress, Toxicity, Inflammation, and Autoimmunity in Relation to Cadmium, Mercury, and Lead in Human Blood: A Pilot Study. Toxics.

[4] Zhao J., Han P.Y., Tian S.N., Shi H.T., He J.H., Xiao C.F. (2019). Polypyrrole/cadmium sulfide hollow fiber with high performance contaminant removal and photocatalytic activity fabricated by layer-by-layer deposition and fiber-sacrifice template approach. J. Colloid. Interf. Sci..

[5] Chen J.Q., Xu Y.M., Han Q., Yao Y.C., Xing H.J., Teng X.H. (2019). Immunosuppression, oxidative stress, and glycometabolism disorder caused by cadmium in common carp (*Cyprinus carpio* L.): Application of transcriptome analysis in risk assessment of environmental contaminant cadmium. J. Hazard. Mater..

[6] Jaehong S., Manish K., Santanu M., Ritusmita G. (2019). Sustainable removal of pernicious arsenic and cadmium by a novel composite of MnO_2_ impregnated alginate beads: A cost-effective approach for wastewater treatment. J. Environ. Manage..

[7] Roy S., Mondal I., Harms K., Chattopadhyay S. (2018). Synthesis, structure, catechol oxidase and phenoxazinone synthase mimicking activity of a manganese(III) Schiff base complex [Mn(HL)_2_(CH_3_OH)_2_][Mn(HL)_2_(N_3_)_2_]. Polyhedron.

[8] Lu Y.Y., Liang X.Q., Niyungeko C., Zhou J.J., Xu J.M., Tian G.M. (2018). A review of the identification and detection of heavy metal ions in the environment by voltammetry. Talanta.

[9] Ruangyuttikarn W., Panyamoon A., Nambunmee K., Honda R., Swaddiwudhipong W., Nishijo M. (2013). Use of the kidney injury molecule-1 as a biomarker for early detection of renal tubular dysfunction in a population chronically exposed to cadmium in the environment. Springerplus.

[10] Kasa N.A., Sel S., Chormey D.S., Bakirdere S. (2019). Determination of cadmium at trace levels in parsley samples by slotted quartz tube-flame atomic absorption spectrometry after preconcentration with cloud point extraction. Measurement.

[11] Zvěřina O., Kuta J., Coufalík P., Kosečková P., Komárek J. (2019). Simultaneous determination of cadmium and iron in different kinds of cereal flakes using high-resolution continuum source atomic absorption spectrometry. Food. Chem..

[12] Tan X.J., Wang Z.M., Wang Z.L. (2018). A Facile Acidic Digestion Method for Cosmetic Lead and Cadmium Determination by an Inductively Coupled Plasma Atomic Emission Spectrometer. J. Appl. Spectrosc..

[13] Azimi S., Es’haghi Z. (2017). A Magnetized Nanoparticle Based Solid-Phase Extraction Procedure Followed by Inductively Coupled Plasma Atomic Emission Spectrometry to Determine Arsenic, Lead and Cadmium in Water, Milk, Indian Rice and Red Tea. Bull. B. Environ. Contam. Tox..

[14] Krata A.A., Wojciechowski M., Kalabun M., Bulska E. (2018). Reference measurements of cadmium and lead contents in candidates for new environmental certified materials by isotope dilution inductively coupled plasma mass spectrometry. Microchem. J..

[15] Gray P.J., Cunningham W. (2019). Inductively Coupled Plasma Collision Cell Quadrupole Mass Spectrometric Determination of Extractible Arsenic, Cadmium, Chromium, Lead, Mercury, and Other Elements in Food Using Microwave-Assisted Digestion: Results from an FDA Interlaboratory Study. J. AOAC Int..

[16] Iammarino M., Taranto A.D., Centonze D. (2017). Determination of Sulphiting Agents in Raw and Processed Meat: Comparison between a Modified Monier-Williams Method and the Direct Analysis by Ion Chromatography with Conductometric Detection. Food. Anal. Method.

[17] Griffin M.J., Kabir K.M., Coyle V.E., Kandjani A.E., Sabri Y.M., Ippolito S.J., Bhargava S.K. (2016). A Nanoengineered Conductometric Device for Accurate Analysis of Elemental Mercury Vapor. Environ. Sci. Technol..

[18] Lisboa T.P., Faria L.V., Matos M.A.C., Matos R.C., Sousa R.A. (2019). Simultaneous determination of cadmium, lead, and copper in the constituent parts of the illegal cigarettes by Square Wave Anodic Stripping Voltammetry. Microchem. J..

[19] Dali M., Zinoubi K., Chrouda A., Abderrahmane S., Cherrad S., Renault N.J. (2018). A biosensor based on fungal soil biomass for electrochemical detection of lead (II) and cadmium (II) by differential pulse anodic stripping voltammetry. J. Electroanal. Chem..

[20] Dahaghin Z., Kilmartin P.A., Mousavi H.Z. (2018). Simultaneous determination of lead(II) and cadmium(II) at a glassy carbon electrode modified with GO@Fe3O4@benzothiazole-2-carboxaldehyde using square wave anodic stripping voltammetry. J. Mol. Liq..

[21] Rosolina S.M., Chambers J.Q., Lee C.W., Xue Z.L. (2015). Direct determination of cadmium and lead in pharmaceutical ingredients using anodic stripping voltammetry in aqueous and DMSO/water solutions. Anal. Chim. Acta.

[22] Tra-ngan S., Siripornadulsil S., Thanwisai L., Siripornadulsil W. (2019). Potential application of a recombinant bacterial strain carrying a groEL promoter as a whole-cell microbial biosensor for detecting bioavailable cadmium. Environ. Technol. Innov..

[23] Ngamdee K., Ngeontae W. (2018). Circular dichroism glucose biosensor based on chiral cadmium sulfide quantum dots. Sens. Actuators B Chem..

[24] Ebrahimi M., Raoof J.B., Ojani R. (2018). Design of an electrochemical DNA-based biosensor for selective determination of cadmium ions using a DNA hybridization indicator. Int. J. Biol. Macromol..

[25] Zeng L.W., Gong J.Y., Rong P.S., Liu C.S., Chen J.H. (2019). A portable and quantitative biosensor for cadmium detection using glucometer as the point-of-use device. Talanta.

[26] Ghenaatian H.R., Fard M.S., Moghadam M.R., Kamath G., Rahmanian M. (2019). Tailoring of graphene quantum dots for toxic heavy metals detection. Appl. Phys. A Mater..

[27] Liu J.Q., Zhang Q.R., Xue W.T., Zhang H.P., Bai Y., Wu L.T., Zhai Z.X., Jin G.H. (2019). Fluorescence Characteristics of Aqueous Synthesized Tin Oxide Quantum Dots for the Detection of Heavy Metal Ions in Contaminated Water. Nanonaterials.

[28] Zhang D.W., Xu Y., Liu Q.L., Xia Z.G. (2018). Encapsulation of CH_3_NH_3_PbBr_3_ Perovskite Quantum Dots in MOF-5 Microcrystals as a Stable Platform for Temperature and Aqueous Heavy Metal Ion Detection. Inorg. Chem..

[29] Siong L.T., Shu J.E., Arundithi A., Kam C.L., Peng C. (2015). Graphene quantum dots functionalized gold nanoparticles for sensitive electrochemical detection of heavy metal ions. Electrochim. Acta.

[30] Pourghobadi Z., Mirahmadpour P., Zare H. (2018). Fluorescent biosensor for the selective determination of dopamine by TGA-capped CdTe quantum dots in human plasma samples. Opt. Mater..

[31] Zare H., Ghalkhani M., Akhavan O., Taghavinia N., Marandi M. (2017). Highly sensitive selective sensing of nickel ions using repeatable fluorescence quenching-emerging of the CdTe quantum dots. Mater. Res. Bull..

[32] Boonmee C., Noipa T., Tuntulani T., Ngeontae W. (2016). Cysteamine capped CdS quantum dots as a fluorescence sensor for the determination of copper ion exploiting fluorescence enhancement and long-wave spectral shifts. Spectrochim. Acta A.

[33] Wu Q., Zhou M., Shi J., Li Q.J., Yang M.Y., Zhang Z.X. (2017). Synthesis of Water-Soluble Ag2S Quantum Dots with Fluorescence in the Second Near-Infrared Window for Turn-On Detection of Zn(II) and Cd(II). Anal. Chem..

[34] Xiao M., Fu Q.Q., Shen H.C., Chen Y., Xiao W., Yan D.G., Tang X.J., Zhong Z.Y., Tang Y. (2018). A turn-on competitive immunochromatographic strips integrated with quantum dots and gold nano-stars for cadmium ion detection. Talanta.

[35] Hu X.Y., Zhu K., Guo Q.S., Liu Y.Q., Ye M.F., Sun Q.J. (2014). Ligand displacement-induced fluorescence switch of quantum dots for ultrasensitive detection of cadmium ions. Anal. Chim. Acta.

[36] Wang Y., Hu X., Wang L., Shang Z., Chao J., Jin W. (2011). A new acridine derivative as a highly selective “off-on” fluorescence chemosensor for Cd^2+^ in aqueous media. Sens. Actuators B Chem..

[37] Xue Y., Zhao H., Wu Z., Li X., He Y., Yuan Z. (2011). Colorimetric detection of Cd^2+^ using gold nanoparticles cofunctionalized with 6-mercaptonicotinic acid and l-cysteine. Analyst.

